# Learning from the Invaders: What Viruses Teach Us about RNA-Based Regulation in Microbes

**DOI:** 10.3390/microorganisms10112106

**Published:** 2022-10-25

**Authors:** L. Peter Sarin

**Affiliations:** RNAcious Laboratory, Molecular and Integrative Biosciences Research Programme, Faculty of Biological and Environmental Sciences, University of Helsinki, 00100 Helsinki, Finland; peter.sarin@helsinki.fi; Tel.: +358-2941-59533

**Keywords:** host–pathogen interactions, infection, viruses, translation, post-transcriptional modification, transfer RNA, bacteria, archaea

## Abstract

Viruses feature an evolutionary shaped minimal genome that is obligately dependent on the cellular transcription and translation machinery for propagation. To suppress host cell immune responses and ensure efficient replication, viruses employ numerous tactics to favor viral gene expression and protein synthesis. This necessitates a carefully balanced network of virus- and host-encoded components, of which the RNA-based regulatory mechanisms have emerged as particularly interesting albeit insufficiently studied, especially in unicellular organisms such as archaea, bacteria, and yeasts. Here, recent advances that further our understanding of RNA-based translation regulation, mainly through post-transcriptional chemical modification of ribonucleosides, codon usage, and (virus-encoded) transfer RNAs, will be discussed in the context of viral infection.

## 1. Introduction

Viruses may rightfully so be considered as the ultimate transmission vectors—they are small and stealthy, omnipresent in nature as each living organism is infected by at least one virus, and they even reside integrated in our genome. Yet, they are not living entities *per se*, as their evolutionary trimmed minimal genome is obligately dependent on the host cell for genome replication and synthesis of viral proteins and assembly of progeny virions [1]. To this end, viruses have developed a baffling array of strategies to commandeer the host cell and its transcription and translation machineries. Lately, significant advances have been made towards understanding how viral infection affects host cell transcription and in particular translation on the molecular level. This has also highlighted the importance of the RNA-based approaches by which viruses regulate cellular functions [2]. Historically, priming the retroviral reverse transcriptase with host initiator transfer RNA (tRNA) constitutes one of the early observations where an RNA component is utilized by the virus to enable replication [3]. Subsequently, post-transcriptional chemical modification of viral messenger RNA (mRNA) transcripts and viral genomic RNA [2], RNA degradation and fragment formation [4], and the utilization of (virus-encoded) tRNAs and tRNA-like structures [5,6,7] are all within the repertoire with which viruses promote their replication and suppress host cell immune responses. Furthermore, a substantial part of our insight on RNA-based regulation stems from studies made on eukaryotes, whereas many microbial model systems are poorly characterized. Given this knowledge gap, further research is likely to reveal fascinating new insights into RNA-based regulation of infection in microbes.

## 2. Tools of the Trade—RNA in the Viroscope

**Figure 1:**
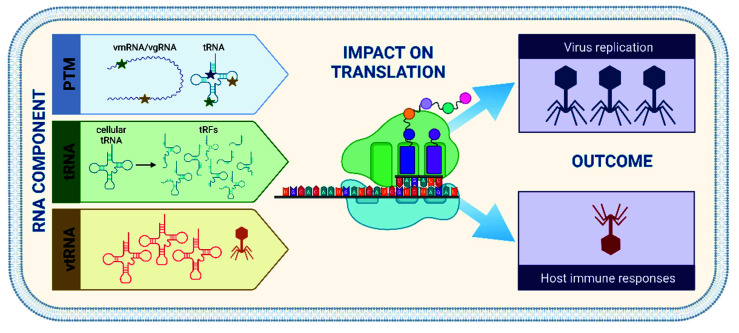
Schematic overview of selected RNA components utilized by viruses to further their replication and suppress host immune responses. Abbreviations: PTM = post-transcriptional chemical modification of ribonucleosides; tRNA = transfer RNA; tRF = tRNA-derived fragment; vgRNA, vmRNA, vtRNA = virus-encoded genomic, messenger, or transfer RNA. Figure created with BioRender.

Throughout evolution, viruses have been—and continue to be—in a constant arms race with their hosts, striving for a balance between efficient viral replication whilst maintaining functionality of the host cell. This is often achieved via an intricate network of viral and host factors. The focus here is on the role of selected RNA components, on what we can learn about protein synthesis control by studying how viruses use these RNA components to interact with the translation machinery (Figure 1).

### 2.1. How Modifications Modulate Cellular Functions

Post-transcriptional chemical modification (PTM) is critical for the structure, function, and metabolism of both coding and non-coding ribonucleosides alike. So far, more than 170 PTMs have been identified [8]. In particular, tRNAs are abundantly decorated with evolutionary conserved modifications containing on average 8-17 PTMs concentrated at key positions; those at the core of the tRNA mostly modulate structural flexibility, whereas the anticodon stem loop (ASL) constitutes a modification hotspot that primarily affects translation rate and fidelity. The importance of tRNA modifications is revealed during stress, such as sudden environmental changes or infection [9]. For example, 2-thiolation reduces proteotoxic stress and regulates the rate of translation [10,11,12], and in hyperthermophiles it also increases tRNA stability [13,14]. Some uridine position 34 (i.e., wobble) modifications have been reported to affect microbial virulence. For instance, loss of 5-carboxymethylaminomethyl (cmnm^5^U_34_) leads to a significant reduction of motility in *Salmonella enterica* sv. *Typhimurium* [15], whereas 2-thiolation deficiency affects thermotolerance in yeast [16] and causes attenuation in *Streptococcus pyogenes* [17]. Furthermore, hypoxia increases uridine 5-oxyacetic acid (cmo^5^U_34_) modification of ${\mathrm{tRNA}}_{\mathrm{Thr}}^{\mathrm{UGU}}$ in *Mycobacterium bovis* BCG, which furthers the translation of transcripts rich in the cognate codon AGC, such as DosR, the master regulator of hypoxic bacteriostasis [18]. PTMs have also been reported to modulate host responses to infection, affecting effector-triggered immunity in plants [19] and numerous innate immune mechanisms for sensing infection in metazoans [2,20,21].

Consequently, it is clear that PTMs are critical regulators of translation in all living organisms [9]. However, viral RNA genomes (vgRNA) and viral mRNA (vmRNA) transcripts are also embellished with evolutionary conserved PTMs, such as methylations, pseudouridine (Ψ), inosine (I), and others. The best studied example is the *N*7-methylguanosine (m^7^G) cap, which was first discovered on vmRNA prior to its identification on cellular mRNA. The 5′-m^7^G cap is noteworthy since it is one of only a handful of modifications that are synthesized by specific virus-encoded RNA modifying enzymes [22]. Aside from the m^7^G cap, other modifications were initially considered to be random additions by overzealous RNA modifying enzymes of the host. It has since been demonstrated that these ‘internal’ PTMs found on viral RNAs have a direct impact on the viral infection cycle by promoting replication, regulating RNA stability, and evading host immune responses [2]. For example, *N*6-methyladenosine (m^6^A) has been reported to (i) mediate nuclear processing and export of vmRNA in simian virus 40 and retroviruses, such as human immunodeficiency virus-1 and Rous sarcoma virus, (ii) aid vmRNA splicing in adenoviruses, and (iii) relocate viral particle formation to viral assembly sites around lipid droplets in hepatitis C virus [23]. In addition, some alphaviruses rely on 5-methylcytidine (m^5^C) modification of their genome to modulate host innate immune responses, whereas m^5^C modification furthers viral gene expression and infectivity in murine leukemia virus [22]. Recently, host acetyltransferase NAT10 catalyzed *N*4-acetylcytidine (ac^4^C) modification of enterovirus 71 internal ribosome entry sites has been shown to boost viral replication, as well as to stabilize viral RNA and enhance binding to the viral RNA-dependent RNA polymerase [24]. Other PTMs, such as Ψ and I, have both been associated with promoting RNA folding [9] and the latter also causes −1 ribosomal frameshifting, which results in the termination of viral protein translation [25]. Moreover, a recent study uncovered that Chikungunya virus infection induces codon-specific reprogramming of the host translation machinery by affecting 5-methylcarboxymethyl (mcm^5^)-containing PTM levels at the ASL of tRNAs, thus favoring the translation of viral transcripts over host mRNAs [26].

As demonstrated above, most of our current knowledge on how viruses utilize PTMs to regulate the host translation machinery is based on eukaryotic model systems. Although PTMs confer equally critical regulatory functions in microbes, comparably little attention has been given to their role during viral infection in prokaryotes or archaea. Studies on the *Escherichia coli* bacteriophage lambda (λ phage) have revealed that host susceptibility to viral infection is governed by a complex network entailing tRNA thiolation, sulfur relay, and ribosomal frameshifting [27,28]. Interestingly, *E. coli* strains deficient in iron-sulfur cluster (ISC)-dependent PTMs, such as 2-thiocytidine (s^2^C) and 2-methylthio (ms^2^), were highly susceptible to λ phage infection, whereas strains deficient in ISC-independent PTMs, such as 2-thiouridine (s^2^U) and 4-thiouridine (s^4^U), were two-fold more resistant to infection than the wildtype strain. This resistance is attributed to loss of 2-thiolation on ${\mathrm{tRNA}}_{\mathrm{Lys}}^{\mathrm{UUU}}$, which alters the rate of −1 ribosomal frameshifting, changing the ratio of proteins synthesized from the λ phage GT region and ultimately, decreasing λ phage production [27]. As exemplified here, viruses utilize an intricate interplay of host mechanisms to further their replication. Given the multitude of microbial species and viruses that infect them, it also underlines the extent of host–virus systems and translational control mechanisms that remain to be explored.

### 2.2. Translation Potluck—Bring Your Own tRNAs

Epitranscriptomic expansion of the genetic code, by modulating PTM abundance on viral transcripts and host non-coding RNAs, is a powerful approach for regulating the fidelity and processivity of translation, as well as influencing the expression of viral proteins [9]. Nonetheless, there are limitations to what can be achieved by PTM modulation alone as some viral genomes display a remarkably divergent codon usage than that of their host. Since viruses are wholly dependent on the host cell translation machinery, this discrepancy in codon usage and availability should manifest as poor translation efficiency of viral transcripts and low viral replication. Astonishingly, many viruses with a high degree of codon mismatch still express their proteins efficiently, which is attributed to virus-encoded tRNAs (vtRNAs), obtained through horizontal gene transfer, that supplement codon availability in the host [5,6]. Translationally active vtRNAs were first reported for bacteriophage T4 [29,30,31], and vtRNAs have since been discovered in evolutionary-diverse viruses throughout all domains of life. Indeed, a study of 13200 viruses found vtRNA genes in as many as 14% of the genomes with the highest prevalence among myoviruses and siphoviruses [32], i.e., head-tailed phages infecting prokaryotes and archaea. Moreover, virulent phages are likely to contain more vtRNA genes than temperate ones due to their higher codon usage bias and difference in GC-content compared to the host [5].

The established viewpoint is that vtRNAs supplement codons that are infrequent or entirely lacking in the host, thus biasing translation towards viral transcripts. This notion holds true for e.g., bacteriophage T4, where all eight vtRNAs contain codons that are infrequent in *E. coli* [33], and many other (jumbo) phages, such as XacN1, where most of its 56 vtRNA genes provide codons that are distinct from the cellular tRNAs encoded by *Xanthomonas citri* [34]. Codon expansion is also thought to allow the virus to overcome host boundaries set by the limitations in the cellular tRNA pool. However, it is not clear to which extent this is a contributing factor as for example cucumber mosaic virus, a generalist virus that infects >1000 plant species, is devoid of vtRNAs although it has multiple tRNA-like structures in its genome [35].

Furthermore, many vtRNA encoding viruses contain only a handful of vtRNA genes representing codons that are not particularly infrequent in the host [32], suggesting that their impact on biasing the cellular codon pool is negligible at best. Hence, are these vtRNAs maintained in the viral genome for a reason, or are they merely remnants of ancient horizontal gene transfer events? One potential answer is that some vtRNAs may not partake in translation, at least not in the canonical sense as amino acid carrying intermediates, but that they might have an altogether different regulatory function. Cues to this effect can be found from eukaryotic cells, where upon physiological stress conditions, such as oxidative stress and high salinity, specific tRNA isoacceptors are processed to yield tRNA-derived fragments (tRFs). These tRFs perform a multitude of regulatory functions affecting e.g., mRNA stability, binding to RNA-binding proteins, translational activation and inactivation, as well as suppression of apoptosis [4]. Interestingly, some viruses also utilize tRFs to further their replication. For example, respiratory syncytial virus infection induces 5′-tRF formation of host ${\mathrm{tRNA}}_{\mathrm{Glu}}^{\mathrm{CUC}}$ and ${\mathrm{tRNA}}_{\mathrm{Gly}}^{\mathrm{CCC}}$, which is thought to further virus replication by suppressing host antiviral responses through a poorly understood *trans*-silencing mechanism [36,37]. Virus-induced 5′-tRFs have also been reported for hepatitis B and C virus [38], but their role remains undetermined. In contrast, some 3′-tRFs may stimulate protein synthesis during stress by invariably binding to ribosomes, thereby generating ‘specialized’ ribosomes with specific translational profiles [39,40]. However, host-induced tRNA degradation can also serve as a suppression mechanism for preventing viral replication. In *E. coli*, bacteriophage T4 infection triggers the latent PrrC anticodon riboendonuclease restriction system, which targets and cleaves host tRNA_Lys_—the most frequently used tRNA in T4 protein synthesis—thereby inhibiting T4 replication [41]. In conclusion, although these functions are mediated by cellular tRFs, it stands to reason that vtRNAs might serve as substrates for targeted degradation and thus, interact with the translation machinery in a similar, multifaceted fashion. Given the prevalence of vtRNAs and their potential regulatory functions, it is surprising that they remain poorly characterized. To date, only a few studies have reported vtRNAs to be expressed and properly charged during infection [5], whereas this information is lacking for the majority of all vtRNA encoding viruses.

## 3. Outlook

There is a wealth of evidence supporting the notion that viruses have mastered the use of cellular components, machineries, and networks to further their replication. Even though many of the RNA-based components and strategies have been known for decades, we still do not fully comprehend or appreciate the intricate interplay between the virus and its host, nor do we know to what extent they are evolutionary conserved. For example, are there specific PTMs that mediate conserved host responses to infection throughout various domains of life? To date, most of our cumulative knowledge stems from eukaryotic model systems and studies on human pathogens. Consequently, the interactions between microbial hosts and their viruses are poorly characterized, with the notable exception of a few well-established laboratory models, such as *E. coli* and its phages. However, fascinating new insights into viral infection, in particular on the role of PTMs and the non-canonical translational function of vtRNAs, are likely to emerge as the vast majority of prokaryotic virus–host systems remain to be explored. Furthermore, the rapidly growing number of known archaeal species and their viruses constitute another treasure-trove, as many are extremophiles with exciting RNA adaptations that enable life in the extremes [42,43], whether in a solar saltern, hot spring, or at the bottom of the ocean next to a thermal vent. Methodological advances for studying PTM dynamics [44,45] and translation [46,47] further push the boundaries for what is feasible and attainable. Taken together, novel species, viruses, and methods will allow us to obtain further insights into the complex network of RNA-mediated transcriptional and translational control during infection. Historically, viruses have been the source for numerous enzymatic tools in molecular biology to manipulate DNA and RNA [1]. Further exploring the function of the poorly characterized vtRNAs might provide new molecular tools that are applicable for directing translation in e.g., synthetic bioproduction systems or as therapeutic agents in a clinical setting. Although future advances cannot be predicted, viruses have surely not surrendered all their secrets, with new cries of “That’s funny…” awaiting to be exclaimed.
